# Dr. J. Marion Sims

**Published:** 1881-05

**Authors:** 


DR. J. MARION SIMS.—We are happy to learn that this distinguished physician and courteous gentleman is convalescing rapidly from his late serious attack of pneumonia, and, it is hoped, will be able soon to resume his important professional duties.

